# A Novel Use for Pepsin

**Published:** 1882-12

**Authors:** 


### A NOVEL USE FOR PEPSIN.

Dr. Hollman (*Nederland Weekblatt*, 18, p. 272) has used an aqueous solution of sixteen grains of pepsin as an injection into the bladder of a patient who had hæmaturia, and in whom a catheter failed to empty the bladder. A few hours later, a dark, viscid, fetid fluid readily escaped through the catheter.—*Ex.*

